# Report of Cases

**Published:** 1890-07

**Authors:** A. W. Griggs

**Affiliations:** West Point, Ga.


### Clinical Department.

### REPORT OF CASES.

BY A. W. GRIGGS, M. D., WEST POINT, GA.

Twelve months ago I visited a female negro child, three months old, which was having convulsions. There was no fever, and the stomach and bowels were reported to be all right. For a time I was at sea in the case, being unable to divine the cause. The child would give a loud shriek and the convulsion would come on and last several minutes. The mother was directed to strip the patient, and upon examination, an umbilical hernia, as large as a medium size hickory nut, was found. This was full of gas and was very easily reduced. A convex silk-covered button was applied and retained by a proper bandage, and though the child had suffered several attacks of the kind, the treatment was entirely successful.

Case 2.—Several years ago I was called to see a male infant, white, age twelve days, which had hemorrhage from the umbilicus. The nurse stated that the cord was slow in dropping off and that the child had bled a little several times, but never so much as now. It was completely blanched, and the temperature was low. Monsel's solution of the subsulphate of iron was used locally with poor results. Plaster of Paris mixed with a little cotton wool, made wet with water, was applied to the umbilicus and held in position until the plaster was moulded to the part and set, then fixed with a bandage. This promised success, but alas for our hopes, the little one died very soon from the loss of blood already sustained.

Case 3.—On March 30th, 1890, I visited a child, a female mulatto, age fourteen days, which was bleeding from the funis, which had not yet come off. I had never seen a funis so late dropping. The child was quite small and very weakly. The funis, as stated, had not ulcerated off, and was a filthy, bloody mass. I ligated it close up and cut it off with scissors. There was still some oozing, and Monsel's solution was used with success. The ligature came away on the second day and the navel looked well. The next morning, word came that the child was bleeding from the left ear. I saw it and cleaned the ear out with warm water and stuffed it with absorbent borated cotton and poured in Monsel's solution, diluted 1-2 with water, and ordered two drops fl. ext. ergot to be given every three hours, or oftener, if the hemorrhage should return. That afternoon the cotton was removed, the ear cleansed and the cotton reapplied without the solution. The next morning there was hemorrhage from the nose. The ergot was increased to five drops with two of the Tr. Ferri chloride. The nose was now washed out with a weak solution of the Monsel's salt. All hemorrhage ceased for three days, when I found it with hemorrhage from the bowels. At the suggestion of my son, I gave it the fl. ext. and black haw. This relieved the bowels, but on the next day its ear received a little scratch on the finna and had to be cauterized. The patient lived on without further hemorrhage, but was found dead in bed with its mother on the fourth day in the morning.

